# Supplementary material for: Seminoma and Embryonal Carcinoma Footprints Identified by Analysis of Integrated Genome-Wide Epigenetic and Expression Profiles of Germ Cell Cancer Cell Lines
Source: PLoS One. 2014 Jun 2;9(6):e98330. doi: 10.1371/journal.pone.0098330 (PMC4041891; doi:10.1371/journal.pone.0098330)
Supplement: File S1 — ZIP file containing DMRforPairs output for significant regions. Please start from the html files. (ZIP) [file pone.0098330.s008.zip › figures/29851.pdf]

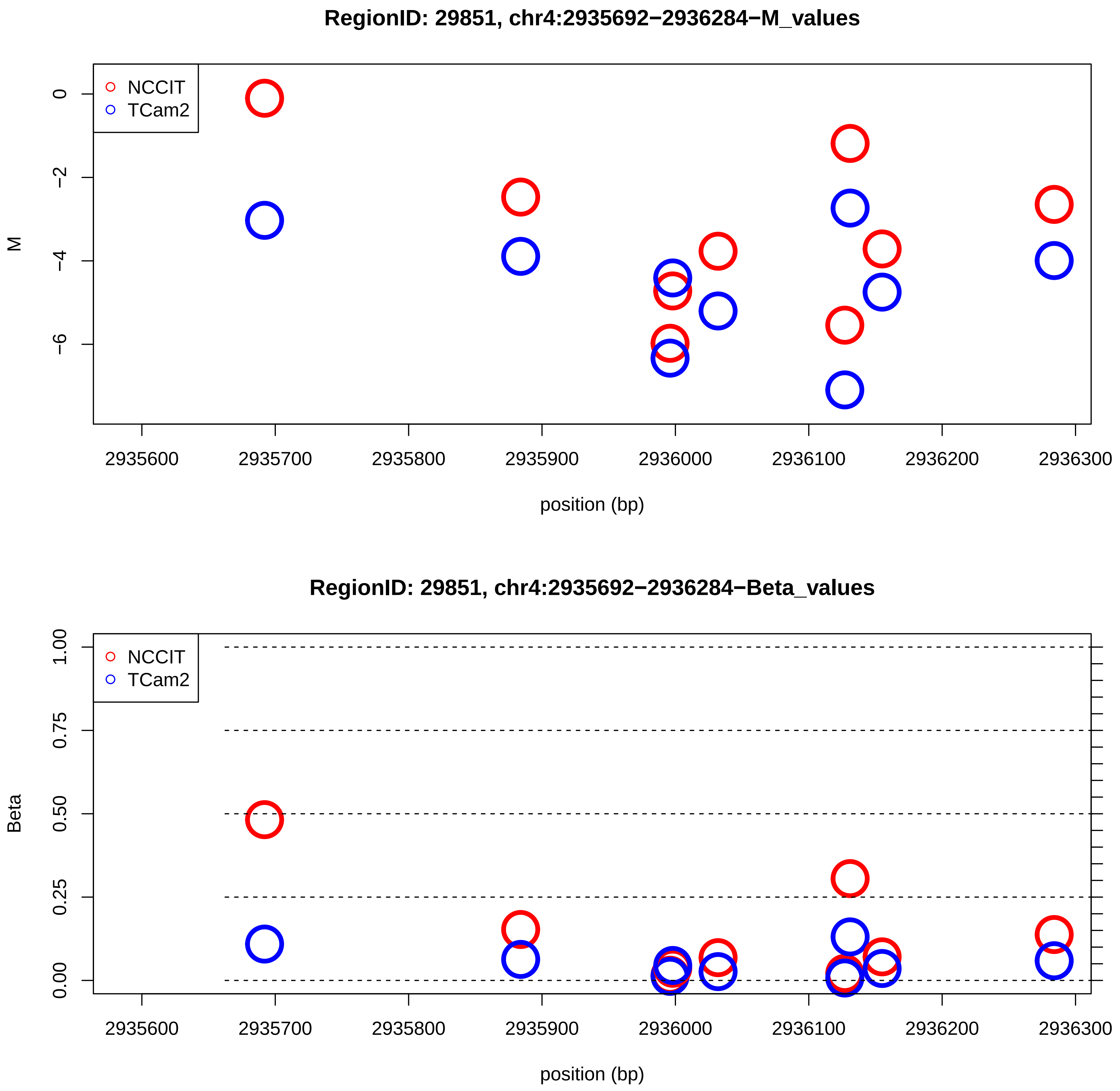
RegionID: 29851, chr4:2935692−2936284−M_values
NCCIT
TCam2
M
0
−2
−4
−6
2935600
2935700
2935800
2935900
2936000
2936100
2936200
2936300
position (bp)
RegionID: 29851, chr4:2935692−2936284−Beta_values
NCCIT
TCam2
Beta
0.00
0.25
0.50
0.75
1.00
2935600
2935700
2935800
2935900
2936000
2936100
2936200
2936300
position (bp)
